# Development of a fed-batch process for a recombinant *Pichia pastoris* Δ*och1* strain expressing a plant peroxidase

**DOI:** 10.1186/s12934-014-0183-3

**Published:** 2015-01-08

**Authors:** Christoph Gmeiner, Amirhossein Saadati, Daniel Maresch, Stanimira Krasteva, Manuela Frank, Friedrich Altmann, Christoph Herwig, Oliver Spadiut

**Affiliations:** Vienna University of Technology, Institute of Chemical Engineering, Research Area Biochemical Engineering, Gumpendorfer Strasse 1a, 1060 Vienna, Austria; Department of Chemistry, University of Natural Resources and Life Sciences, Vienna, Austria

**Keywords:** *Pichia pastoris*, Glycosylation, OCH1, Horseradish peroxidase, Bioreactor cultivation, Fed-batch, Design of Experiments

## Abstract

*Pichia pastoris* is a prominent host for recombinant protein production, amongst other things due to its capability of glycosylation. However, N-linked glycans on recombinant proteins get hypermannosylated, causing problems in subsequent unit operations and medical applications. Hypermannosylation is triggered by an α-1,6-mannosyltransferase called OCH1. In a recent study, we knocked out OCH1 in a recombinant *P. pastoris* CBS7435 Mut^S^ strain (Δ*och1*) expressing the biopharmaceutically relevant enzyme horseradish peroxidase. We characterized the strain in the controlled environment of a bioreactor in dynamic batch cultivations and identified the strain to be physiologically impaired. We faced cell cluster formation, cell lysis and uncontrollable foam formation.

In the present study, we investigated the effects of the 3 process parameters temperature, pH and dissolved oxygen concentration on 1) cell physiology, 2) cell morphology, 3) cell lysis, 4) productivity and 5) product purity of the recombinant Δ*och1* strain in a multivariate manner. Cultivation at 30°C resulted in low specific methanol uptake during adaptation and the risk of methanol accumulation during cultivation. Cell cluster formation was a function of the C-source rather than process parameters and went along with cell lysis. In terms of productivity and product purity a temperature of 20°C was highly beneficial. In summary, we determined cultivation conditions for a recombinant *P. pastoris* Δ*och1* strain allowing high productivity and product purity.

**Electronic supplementary material:**

The online version of this article (doi:10.1186/s12934-014-0183-3) contains supplementary material, which is available to authorized users.

## Introduction

The methylotrophic yeast *Pichia pastoris* is an attractive host for the recombinant production of proteins and biopharmaceuticals (e.g. [1-3]). It can grow on inexpensive media to high cell densities [1], numerous molecular manipulation tools are available [4] and high production titers are possible [5,6]. Due to the capacity of performing posttranslational modifications, like glycosylation, *P. pastoris* is attractive for the production of eukaryotic proteins (e.g. [3,7-10]). However, the glycosylation capacity of this yeast also is a curse: native glycosyltransferases recognize the aminoacid motif N-X-S/T and link N-glycans to the asparagine [11,12]. In contrast to mammalians, however, no trimming reactions of the attached glycans happen, but the glycans are further extended, a phenomenon known as hyperglycosylation [13]. The first reaction of this cascade is catalyzed by an α-1,6-mannosyltransferase (OCH1) localized in the Golgi apparatus [14,15]. Hyperglycosylation describes a huge problem since not only the physico-chemical properties of the target protein get masked leading to difficulties in the downstream process [16], but also yeast derived glycans are not compatible with the human organism and can cause immunogenic reactions [17]. Consequently, there have been numerous attempts to manipulate the native glycosylation machinery of *P. pastoris* (e.g. [18-22]). In a recent study, we deleted OCH1 in a recombinant *P. pastoris* strain (Δ*och1*) and physiologically characterized the strain in the controlled environment of a bioreactor [23]. We purified the recombinant product horseradish peroxidase (EC 1.11.1.7; HRP; e.g. [24]) and analyzed catalytic constants, thermal stability as well as protein glycosylation. Although the Δ*och1* strain produced the recombinant protein with shorter glycans of considerably increased homogeneity, the strain was physiologically impaired and thus hard to cultivate. We faced cell cluster formation, cell lysis and uncontrollable foam formation [25,26].

In the present study, we investigated the effects of the 3 process parameters temperature, pH and dissolved oxygen concentration (dO_2_) on 1) cell physiology, 2) cell morphology, 3) cell lysis, 4) productivity and 5) product purity in a multivariate manner to identify fed-batch operating conditions for the recombinant Δ*och1* strain which give both high productivity and product purity, and hamper methanol accumulation as well as cell lysis and consequent foam formation.

## Material and methods

### Microorganism

A *P. pastoris* CBS7435 Mut^S^ Δ*och1* strain carrying the gene coding for the HRP isoenzyme A2A was provided by Prof. Anton Glieder (University of Technology, Graz, Austria). Strain generation and isoenzyme characteristics were described previously [23,27]. A recombinant *P. pastoris* CBS7435 Mut^S^ strain with intact OCH1 expressing HRP A2A, hereafter called wildtype OCH1 strain, was included as reference.

### Design of experiments

A 2^3^-level full factorial screening approach with 2 centre points was set up with the program MODDE (Umetrics, Sweden) to explore the influence of the 3 factors temperature (20-30°C), pH (5.0-7.0) and dO_2_ (10–30%) as well as their linear interactions on different response parameters resulting in a total of 10 fed-batch cultivations (Table 1). We chose the limits for temperature with 20-30°C, since this temperature range is reported for yeasts (e.g. [28-31]). For pH we investigated values between pH 5.0 and 7.0 (e.g. [32]), since *P. pastoris* does not grow well at more acidic or alkaline conditions and also HRP exhibits high stability in this pH range [16]. Finally, we investigated dO_2_ levels between 10–30%, which is again a range which had been used for *P. pastoris* before (e.g. [30,32,33]).

**Table 1:** **Experimental plan for the multivariate analysis of the 3 factors temperature, pH and dO**_2_ **and their effects on different response parameters**

**Cultivation**	**Run order**	**Temperature [°C]**	**pH**	**dO**_**2**_ **[%]**
DoE1	9	20	5	30
DoE2	1	20	5	10
DoE3	8	30	5	30
DoE4	2	30	7	30
DoE5	6	30	7	10
DoE6	3	25	6	20
DoE7	10	20	7	10
DoE8	4	20	7	30
DoE9	7	25	6	20
DoE10	5	30	5	10

We analyzed the effects of the 3 factors on 1) strain physiology (specific substrate uptake rate during methanol adaptation (q_s MeOH adapt_), methanol accumulation during fed-batch (MeOH_accum_), biomass yield (Y_X/S_) and CO_2_ yield (Y_CO2/S_)), 2) strain morphology (cell size distribution), 3) cell lysis (extracellular DNA content), 4) productivity (space-time-yield STY [U∙L^−1^∙h^−1^], specific productivity q_p_ [U∙g^−1^∙h^−1^]) and 5) product purity (defined as the amount of target protein in relation to the amount of total extracellular protein [U∙mg^−1^]).

### Bioreactor cultivations

The *P. pastoris* Δ*och1* strain expressing HRP isoenzyme A2A was cultivated in the controlled environment of a bioreactor. Batch and fed-batch phase were performed on glycerol, followed by a methanol adaptation pulse. Afterwards, a methanol fed-batch with a controlled feed rate corresponding to a certain specific substrate uptake rate of methanol (q_s MeOH_) was done.

#### Culture media

Precultures were done in yeast nitrogen base medium (YNBM; 0.1 M potassium phosphate buffer pH 6.0, 3.4 g · L^−1^ YNB w/o amino acids and ammonia sulfate, 10 g · L^−1^ (NH_4_)_2_SO_4_, 400 mg · L^−1^ biotin, 20 g · L^−1^ glucose). Zeocine was added at a concentration of 100 μg∙L^−1^.

Batch and fed-batch cultivations were performed in 2-fold concentrated basal salt medium (BSM; 21.6 mL · L^−1^ 85% phosphoric acid, 0.36 g∙L^−1^ CaSO_4_ · 2H_2_O, 27.24 g∙L^−1^ K_2_SO_4_, 4.48 g∙L^−1^ MgSO_4_ · 7H_2_O, 8.26 g∙L^−1^ KOH, 0.3 mL∙L^−1^ Antifoam Struktol J650, 4.35 mL∙L^−1^ PTM1, NH_4_OH as N-source). Trace element solution (PTM1) was made of 6.0 g · L^−1^ CuSO_4_ · 5H_2_O, 0.08 g · L^−1^ NaI, 3.0 g · L^−1^ MnSO_4_ · H_2_O, 0.2 g · L^−1^ Na_2_MoO_4_ · 2H_2_O, 0.02 g · L^−1^ H_3_BO_3_, 0.5 g · L^−1^ CoCl_2_, 20.0 g · L^−1^ ZnCl_2_, 65.0 g · L^−1^ FeSO_4_ · 7H_2_O, 0.2 g · L^−1^ biotin, 5 mL · L^−1^ H_2_SO_4_. Induction was carried out in presence of the heme-precursor Δ-aminolevulinic acid at a final concentration of 1 mM. The concentration of the base NH_4_OH was determined by titration with 0.25 M potassium hydrogen phthalate.

#### Preculture

Frozen stocks (−80°C) were cultivated in 100 mL YNBM-Zeocine in 1,000 mL shake flasks at 30°C and 230 rpm for 48 h. Then, the preculture was transferred aseptically to the culture vessel. The inoculum volume was 10% of the final starting volume.

#### Batch and non-induced fed-batch

Batch cultivations were carried out in a 3 L working volume glass bioreactor (Infors, Switzerland). Basal salt medium was sterilized in the bioreactor and pH was adjusted to pH 6.0, which corresponds to the centre point of the subsequent DoE, by concentrated NH_4_OH solution after autoclaving. Sterile filtered trace elements were transferred to the reactor. Dissolved oxygen (dO_2_) was measured with a sterilizable fluorescence dissolved oxygen electrode (Visiferm DO425, Hamilton, Germany). The pH was measured with a sterilizable electrode (Easyferm™, Hamilton, Switzerland) and maintained constant with a PID controller using NH_4_OH solution (2 to 3 M). Base consumption was determined gravimetrically. Cultivation temperature was set to 30°C and agitation was fixed to 900 rpm. The culture was aerated with 1.0 vvm dried air and off-gas of the culture was measured by using an infrared cell for CO_2_ and a paramagnetic cell for O_2_ concentration (Servomax, Switzerland). Temperature, pH, dO_2_, agitation as well as CO_2_ and O_2_ in the off-gas were measured online and logged in a process information management system (PIMS; Lucullus, Biospectra, Switzerland). After the complete consumption of the substrate glycerol, indicated by an increase of dO_2_ and a drop in off-gas activity, an exponential fed-batch phase on glycerol with a specific growth rate of μ = 0.08 h^−1^ was performed. Based on the amount of glycerol used in the batch and the biomass yield on glycerol of Y_X/S_ = 0.47 g∙g^−1^, we calculated the biomass concentration after the batch phase. The feed rate was then determined by equations 1 and 2 and controlled by the PIMS. The fed-batch on glycerol was stopped when the reactor volume reached 2.2 L.

1 $$ {\boldsymbol{F}}_0 = \frac{\boldsymbol{X}\cdot \boldsymbol{V}\cdot \boldsymbol{\upmu} \cdot {\boldsymbol{\delta}}_{\boldsymbol{F}\boldsymbol{eed}}}{{\boldsymbol{Y}}_{\frac{\boldsymbol{X}}{\boldsymbol{S}}}\cdot {\boldsymbol{c}}_{\boldsymbol{F}\boldsymbol{eed}}} $$

2 $$ \boldsymbol{F} = {\boldsymbol{F}}_0\cdot {\boldsymbol{e}}^{\left(\boldsymbol{\mu} \cdot \boldsymbol{t}\right)} $$

F_0_ = initial feed rate [g∙h^−1^]; X = calculated biomass concentration [g∙L^−1^]; V = volume in the bioreactor [L]; μ = specific growth rate [h^−1^]; δ_Feed_ = density glycerol feed [g∙L^−1^]; Y_X/S_ = biomass yield; c_Feed_ = concentration feed [g∙L^−1^]; F = calculated feed rate [g∙h^−1^]; e, Euler constant; t = time [h]

Before fed-batch experiments, a single batch cultivation with dynamic methanol pulses was performed to determine q_smax MeOH_ at 20°C, 25°C and 30°C [25,26,34]. After complete consumption of glycerol, a methanol adaptation pulse (supplemented with 12 mL · L^−1^ PTM1) of a final concentration of 0.5% (v/v) was conducted. Following pulses were performed with 1% methanol (v/v). At least 3 consecutive methanol pulses were analyzed at each temperature. For each pulse, at least two samples were taken to determine the concentrations of substrate and product, as well as dry cell weight to calculate specific rates and yields. Furthermore, biomass was used to determine the correlation between dry cell weight (DCW) and optical density at 600 nm (OD_600_). The DCW of diluted samples (1:2, 1:4, 1:6, 1:8 and 1:10) was plotted against the corresponding OD_600_ values (Genesys 20; Thermo Scientific, Austria) resulting in a so-called α-factor, which was used for biomass calculation in subsequent fed-batch cultivations (equation 3).

3 $$ \mathbf{X} = \mathbf{O}{\mathbf{D}}_{600} \cdot \boldsymbol{\upalpha} $$

X = calculated biomass [g∙L^−1^]; OD_600_ = optical density at 600 nm; α = correlation factor between DCW and OD_600_ value.

#### Induction

After glycerol depletion, temperature, pH and dO_2_ were changed according to Table 1. To control dO_2_, a PID controller was implemented regulating dO_2_ by aeration and not by stirrer speed. Thus, potential influences on cell cluster formation were omitted. After parameters reached the target values, a 0.5% (v/v) methanol adaptation pulse was applied. Concomitantly, the heme-precursor Δ-aminolevulinic acid was aseptically added at a final concentration of 1 mM. When the culture was adapted to methanol, indicated by an increase of dO_2_ and a drop in off-gas activity, a methanol fed-batch at a feed rate corresponding to a q_s MeOH_ of 0.2 mmol∙g^−1^∙h^−1^ was performed for around 100 hours. The feed rate was determined by equation 4 and controlled by the PIMS.

4 $$ \boldsymbol{F} = \frac{\boldsymbol{X}\cdot \boldsymbol{V}\cdot {\mathbf{q}}_{\mathbf{s},\mathbf{MeOH}}\cdot {\boldsymbol{\delta}}_{\boldsymbol{Feed}}}{{\boldsymbol{c}}_{\boldsymbol{Feed}}} $$

F = calculated feed rate [g∙h^−1^]; X = biomass concentration calculated from OD_600_ [g∙L^−1^]; V = volume in the bioreactor [L]; q_s MeOH_ = specific methanol uptake rate [mmol∙g^−1^∙h^−1^]; C_Feed_ = density of methanol feed [g∙L^−1^]; c_Feed_ = concentration of methanol feed [g∙L^−1^].

#### Sample analysis

Dry cell weight was determined by centrifugation of 5 mL culture broth (5,000 rpm, 4°C, 10 min) in a laboratory centrifuge (Sigma 4K15, rotor 11156), washing the pellet with 5 mL deionized water and subsequent drying at 105°C to a constant weight in an oven. The enzymatic activity of HRP was measured using an ABTS assay in a CuBiAn XC enzymatic robot (Innovatis, Germany). Ten μl of sample were mixed with 140 μl 1 mM ABTS solution (50 mM KH_2_PO_4_, pH 6.5). The reaction mixture was incubated at 37°C for 5 min before the reaction was started by the addition of 20 μl 0.078% H_2_O_2_ (v/v). Changes in absorbance at 415 nm were measured for 80 seconds and rates were calculated. The standard curve was prepared using a commercially available HRP preparation (Type VI-A, Sigma-Aldrich, USA) in the range from 0.02 to 2.0 U · ml^−1^. Protein concentrations were determined at 595 nm using the Bradford Protein Assay Kit (Bio-Rad Laboratories GmbH, Austria) with bovine serum albumin as standard. Extracellular DNA content was measured by the Nanodrop 1000 device (ThermoScientific, Austria). Concentrations of methanol and potential metabolites were determined in cell-free samples by HPLC (Agilent Technologies, USA) equipped with an ion-exchange column (Supelcogel C-610H Sigma-Aldrich, USA) and a refractive index detector (Agilent Technologies, USA). The mobile phase was 0.1% H_3_PO_4_ with a constant flow rate of 0.5 mL · min^−1^ and the system was run isocratically at 30°C. All measurements were done in duplicates.

#### Strain morphology

Changes in the morphology of the recombinant *P. pastoris* Δ*och1* strain during the bioprocess were monitored by a Malvern Mastersizer 2000, measuring the cell size distribution in all samples. Frozen cell pellets (stored at −20°C) with a biomass concentration of 10–20 g∙L^−1^ were thawed and resuspended in 10 mL deionized water. After dispersing the samples with module Hydro2000S, 6 to 20 drops of cell suspension were dripped into the water tank of the Malvern Mastersizer 2000 until the laser diffraction reached a value between 10–12%. Samples were analyzed between 0 – 10,000 μm. Furthermore, all samples were analyzed microscopically using a Zeiss Epifluorescence Axio Observer Z1 deconvolution microscope (Carl Zeiss, Germany) equipped with a LD Plan-Neofluar 63x objective (+10x ocular) and the LED illumination system Colibri.

### Electrophoresis

Electrophoresis was done with aliquots of supernatants obtained at the end of the cultivation. SDS-PAGE was performed using a 6% stacking gel and a 12% separating gel in 1x Tris-glycine buffer. Gels were run in the vertical electrophoresis Mini-PROTEAN Tetra Cell apparatus (Biorad; Austria) at 150 V for about 2 h. Gels were stained with Coomassie blue. The protein mass standard used was the PageRuler Prestained Ladder (Fermentas; Austria).

### Protein identification and glycopeptide analysis by LC-ESI-MS

Relevant protein bands were cut out and digested in gel. S-alkylation with iodoacetamide and digestion with sequencing grade modified trypsin (Promega) were performed. The peptide mixture was analysed using a Dionex Ultimate 3000 system directly linked to a QTOF instrument (maXis 4G ETD, Bruker) equipped with the standard ESI source in the positive ion, DDA mode (= switching to MSMS mode for eluting peaks). MS-scans were recorded (range: 150–2200 Da) and the 6 highest peaks were selected for fragmentation. Instrument calibration was performed using ESIcalibration mixture (Agilent). For separation of the peptides a Thermo BioBasic C18 separation column (5 μm particle size, 150°0.360 mm) was used. A gradient from 95% solvent A and 5% solvent B (Solvent A: 65 mM ammonium formiate buffer, B: 100% ACCN) to 32% B in 45 min was applied, followed by a 15 min gradient from 32% B to 75% B, at a flow rate of 6 μL∙min^−1^. The analysis files were converted using Data Analysis 4.0 (Bruker) to XML files, which are suitable to perform MS/MS ion searches with MASCOT (embedded in ProteinScape 3.0, Bruker) for protein identification. Only proteins identified with at least 2 peptides with a protein score higher than 80 were accepted. For searches the SwissProt database was used.

## Results and discussion

In the present study we developed a fed-batch bioprocess for a recombinant *P. pastoris* Δ*och1* strain. We analyzed the effects of 3 process parameters (temperature, pH and dO_2_) on strain physiology and morphology [23], as well as on productivity and product purity in a multivariate manner.

### Dynamic batch cultivation to determine q_s max MeOH_

We performed a dynamic batch cultivation with methanol pulses to determine the maximum specific methanol uptake rate (q_s max MeOH_) of the Δ*och1* strain at 20°C, 25°C and 30°C [25,26]. The average q_s max MeOH_ determined for at least 3 consecutive 1% (v/v) methanol pulses were 0.43 mmol∙g^−1^∙h^−1^ at 30°C, 0.74 mmol∙g^−1^∙h^−1^ at 25°C and 0.85 mmol∙g^−1^∙h^−1^ at 20°C. Interestingly, q_s max MeOH_ was strongly temperature dependent. At 20°C, cells specifically consumed the double amount of methanol per time compared to 30°C. In previous studies with different microorganisms it was shown that substrate uptake usually declines with lower temperature [35-37]. Thus, we also analyzed a reference strain with intact OCH1, hereafter referred to as wildtype OCH1 strain, and indeed observed decreasing q_s MeOH_ with decreasing temperature, namely 1.30 mmol∙g^−1^∙h^−1^ at 28°C, 1.20 mmol∙g^−1^∙h^−1^ at 24°C and 0.95 mmol∙g^−1^∙h^−1^ at 20°C. So far, we do not have a physiological explanation for the opposite behaviour of the recombinant Δ*och1* strain. However, since the subsequent DoE covered a range from 20-30°C we designed the fed-batch strategy for the recombinant Δ*och1* strain in a way to constantly feed at a rate corresponding to a rather low q_s MeOH_ = 0.2 mmol∙g^−1^∙h^−1^. This was less than half of the lowest q_s max MeOH_ determined in the dynamic batch experiment, and thus guaranteed a certain safety margin to avoid methanol accumulation.

### DoE fed-batch cultivations

**Figure 1:**
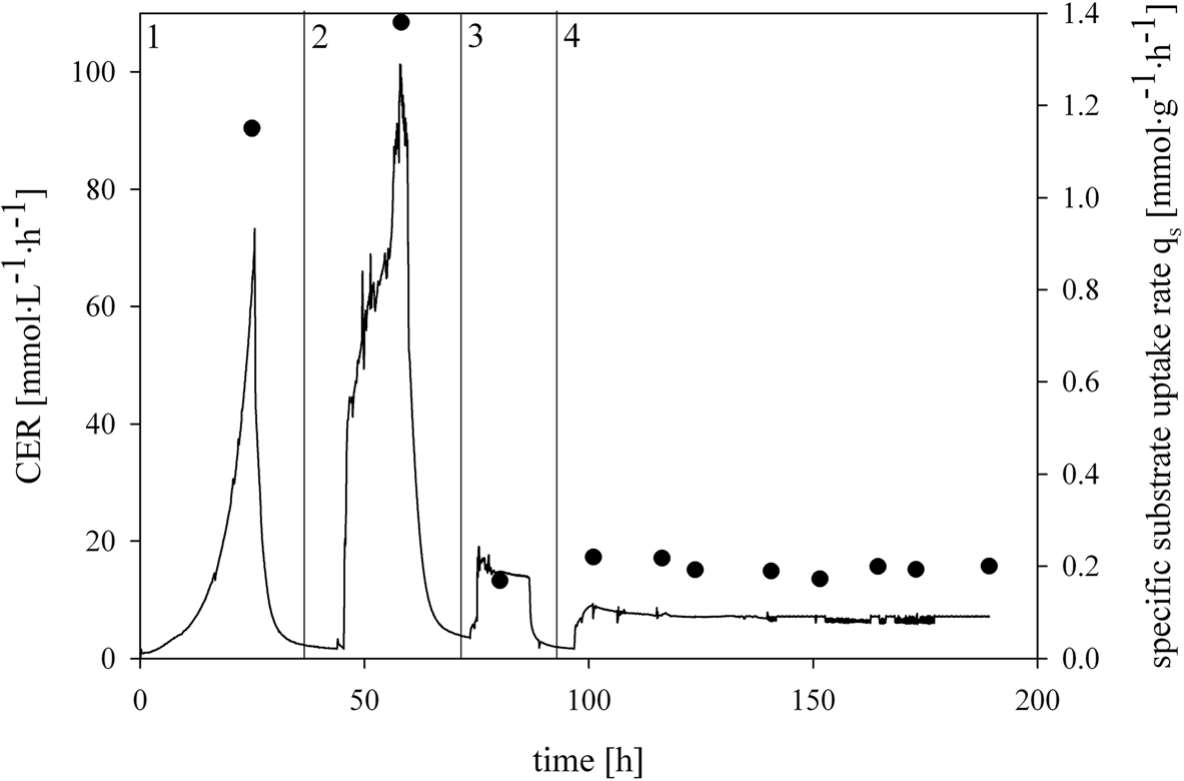
**Bioreactor cultivation DoE6.** Black line, carbon dioxide evolution rate (CER) depicting metabolic activity; black dot, specific substrate uptake rate (q_s_). All cultivations ran in four phases: 1, batch on glycerol; 2, non-induced fed-batch on glycerol; 3, methanol adaptation pulse; 4, fed-batch on methanol.

We analyzed the effects of the 3 process parameters temperature, pH and dO_2_ on 1) strain physiology, 2) strain morphology, 3) cell lysis, 4) productivity and 5) product purity of the recombinant Δ*och1* strain. The goal of this multivariate study was to find operating conditions in fed-batch mode which give high productivity and product purity and concomitantly hamper methanol accumulation as well as cell lysis and consequent foam formation. Therefore, we conducted 10 fed-batch experiments in the controlled environment of a bioreactor (Table 1). The batch and the non-induced fed-batch were always performed under the same conditions. Only prior methanol adaptation (*i.e.* the phase during the *Pichia pastoris* cells get adapted to methanol) process parameters were changed. The carbon dioxide evolution rate (CER) depicting such a bioprocess is exemplarily shown for cultivation DoE6 in Figure 1.

#### Effects on strain physiology

By frequent sampling and subsequent analyses, we physiologically characterized the recombinant Δ*och1* strain. The most relevant strain characteristic parameters are summarized in Table 2. The specific glycerol uptake rate in the batch phase (q_s__Gly batch_) was very similar in all cultivations. Only during the induction phase strain characteristic parameters changed. The q_s MeOH_ in the different fed-batches was close to the set value of 0.2 mmol∙g^−1^∙h^−1^, underlining the validity of the feeding strategy. Furthermore, closing C-balances underline the reliability of the data (Table 2).

**Table 2:** **Strain characteristic parameters of the recombinant** ***P. pastoris*** **Δ** ***och1*** **strain induced under different conditions**

**Cultivation**	**q**_**s**__**Gly batch**_	**q**_**s MeOH adapt**_	**q**_**s MeOH fed-batch**_	**MeOH**_**accum**_	**Y**_**CO2/S**_	**Y**_**X/S**_	**C-balance**
	**[mmol∙g** ^**−1**^ **∙h** ^**−1**^ **]**	**[mmol∙g** ^**−1**^ **∙h** ^**−1**^ **]**	**[mmol∙g** ^**−1**^ **∙h** ^**−1**^ **]**	**[g∙L** ^**−1**^ **]**	**[C-mol∙C-mol** ^**−1**^ **]**	**[C-mol∙C-mol** ^**−1**^ **]**	
DoE1	1.42	0.23	0.20	-	0.80	0.21	1.01
DoE2	1.40	0.26	0.26	-	0.80	0.20	1.00
DoE3	1.38	0.08	0.18	0.68	0.82	0.23	1.05
DoE4	1.28	0.09	0.16	4.90	0.82	0.24	1.06
DoE5	1.26	0.16	0.21	-	0.83	0.26	1.09
DoE6	1.25	0.17	0.20	-	0.75	0.32	1.07
DoE7	1.39	0.16	0.20	-	0.71	0.29	1.00
DoE8	1.40	0.18	0.24	-	0.69	0.33	1.02
DoE9	1.42	0.15	0.20	-	0.75	0.28	1.03
DoE10	1.42	0.16	0.21	0.20	0.80	0.27	1.07

##### Specific substrate uptake rate during methanol adaptation (q_s MeOH adapt_)

**Figure 2:**
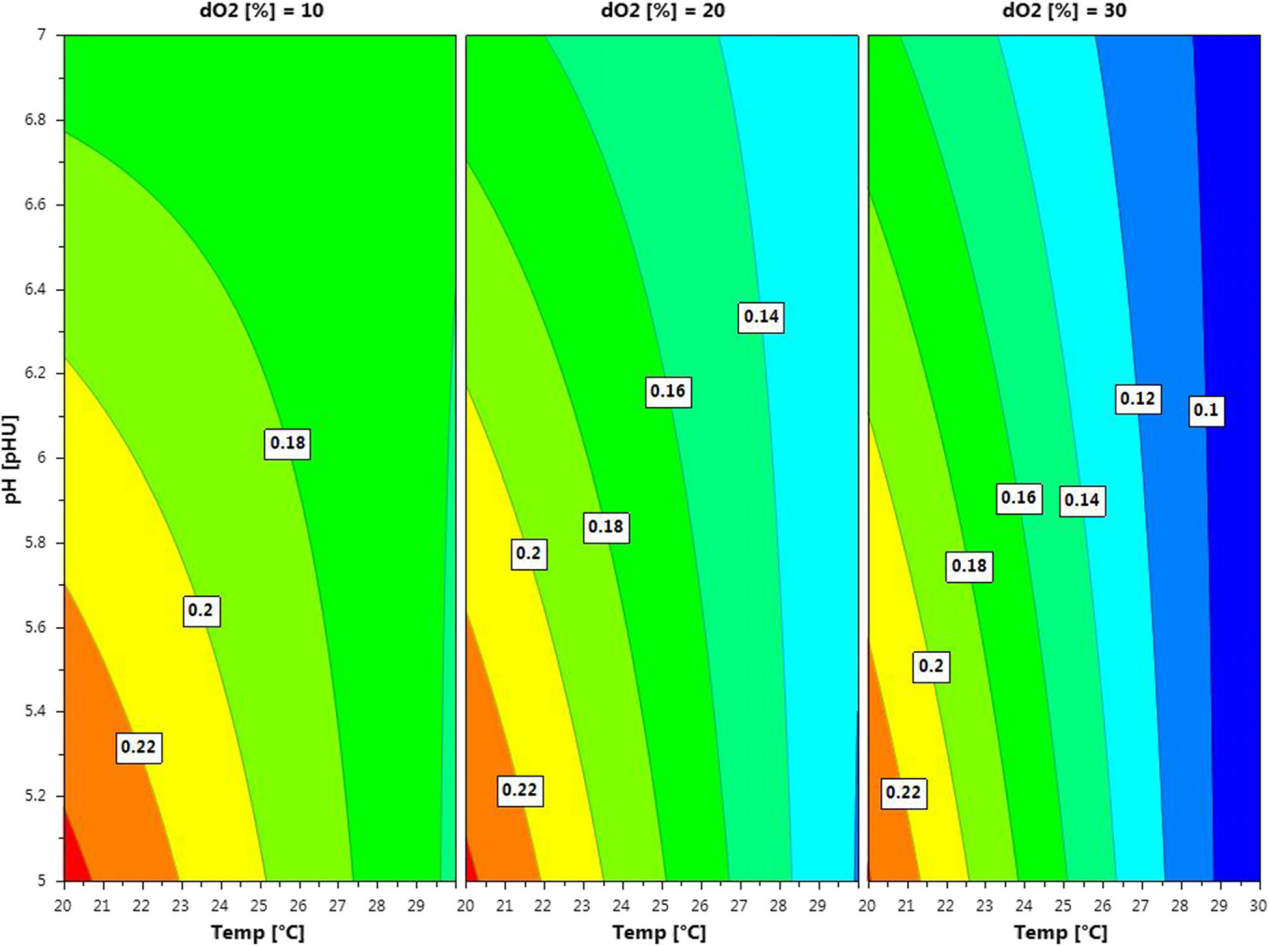
**Contour plot showing the specific substrate uptake rate for methanol during adaptation (q**_s MeOH adapt_ **) in dependence on the 3 process parameters temperature, pH and dO**_2_ **.**

As shown in Figure 2, all 3 factors significantly affected q_s MeOH adapt_ with temperature being the most significant one (p-values: temperature = 0.0011, pH = 0.0406, dO_2_ = 0.0055). The summary of fit plot indicated a valid model (R2 = 0.99, Q2 = 0.90, Model Validity = 0.95, Reproducibility = 0.95). At 30°C and a dO_2_ of 30% the recombinant Δ*och1* strain only consumed 0.1 mmol∙g^−1^∙h^−1^, whereas this value was more than doubled at 20°C. Interestingly, the wildtype OCH1 strain showed the opposite as at 30°C 0.37 mmol∙g^−1^∙h^−1^ methanol were consumed but only half was consumed at 20°C (0.18 mmol∙g^−1^∙h^−1^). Summarizing, the lower the temperature, pH and dO_2_, the higher was q_s MeOH adapt_ for the recombinant Δ*och1* strain (Figure 2).

##### Methanol accumulation during fed-batch (MeOH_accum_)

During induction the feed rate was regulated to correspond to a constant q_s MeOH_ = 0.2 mmol∙g^−1^∙h^−1^, which was 2-fold lower than the lowest q_s max MeOH_ at 30°C determined in the dynamic batch experiment. However, methanol still accumulated in cultivations DoE3, DoE4 and DoE10. For DoE3 methanol accumulation set on after around 80 h and for DoE10 after around 70 h of induction, whereas for DoE4 methanol accumulated right from the start. In Table 2 methanol concentrations at the end of cultivation are shown. Methanol only accumulated in cultivations at 30°C which is in agreement with both the results from the dynamic batch cultivation at 30°C where q_s max_ for methanol was the lowest, as well as with the low q_s MeOH adapt_ in fed batch cultivations at 30°C (Figure 2). However, we could not identify a significant factor causing this phenomenon since in DoE5 (30°C, pH 7.0, 10% dO_2_) no methanol accumulated (Table 2). Apparently, induction at 30°C favours methanol accumulation over time, a phenomenon which was observed before [23]. However, using the current DoE screening approach based on only 3 factors and the underlying linear regression model, we could not identify significant factors and their interactions causing methanol accumulation. Apparently, a temperature of 30°C favours changes in cell physiology and a reduction of methanol uptake over time which is why a lower cultivation temperature of 20°C for fed-batch production processes with this Δ*och1* strain is highly recommended. In contrast, the wildtype OCH1 strain can be cultivated at 30°C without experiencing changes in methanol uptake.

##### Yields

We investigated the influence of the 3 process parameters on both biomass yield (Y_X/S_) and CO_2_ yield (Y_CO2/S_). However, yields were not affected by varying the 3 process parameters in the design space (Table 2).

#### Effects on strain morphology

In a previous study, we had observed cluster formation of the recombinant Δ*och1* strain during batch cultivation [23]. To analyze this phenomenon in more detail and monitor it in the different phases of a fed-batch, samples taken during the different fed-batch cultivations were analyzed for cell size distribution in a Malvern Mastersizer. The cell size of an average *P. pastoris* cell lies between 4–6 μm [38]. Of course, budding cells, where two or more daughter cells are still attached to the mother cell, are larger, which is why we categorized all signals < 15 μm as single and budding cells and all signals > 15 μm as cell clusters. We were especially interested in comparing the samples taken after batch and after non-induced fed-batch, which were both performed on glycerol, with the sample taken at the end of the induction phase on methanol (Table 3). We performed these measurements for all bioreactor cultivations, calculated mean values and standard deviations and compared the results with a wildtype OCH1 strain which was cultivated in fed-batch mode at 25°C, pH 6.0 and 20% dO_2_, corresponding to the DoE centre point (Table 1).

**Table 3:** **Cell-size distribution at different time points of the fed-batch cultivation. Single and budding cells were categorized with a size of < 15 μm, whereas cell clusters were classified to be > 15 μm**

**Cultivation**	**Size distribution after batch [%]**		**Size distribution after non-induced fed-batch [%]**		**Size distribution after induced fed-batch [%]**	
	**<15 μm**	**>15 μm**	**< 15 μm**	**> 15 μm**	**< 15 μm**	**> 15 μm**
DoE1	53.0	47.0	41.5	58.5	79.8	20.2
DoE2	56.8	43.2	64.8	35.2	79.9	20.1
DoE3	54.7	45.3	60.5	39.5	64.7	35.3
DoE4	38.3	61.7	57.1	42.9	80.6	19.4
DoE5	53.8	46.2	74.8	25.2	73.3	26.7
DoE6	55.7	44.3	70.9	29.1	81.0	19.0
DoE7	36.3	63.7	42.5	57.5	75.8	24.2
DoE8	40.8	59.2	47.0	53.0	71.0	29.0
DoE9	49.9	50.1	69.5	30.5	90.4	9.6
DoE10	48.2	51.8	69.4	30.6	89.1	10.9
average	48.8 ± 7.6	51.3 ± 7.6	59.8 ± 12.3	40.2 ± 12.3	78.6 ± 7.8	21.4 ± 7.8
wildtype OCH1 strain	87.5	12.5	87.3	12.7	86.5	13.5

**Figure 3:**
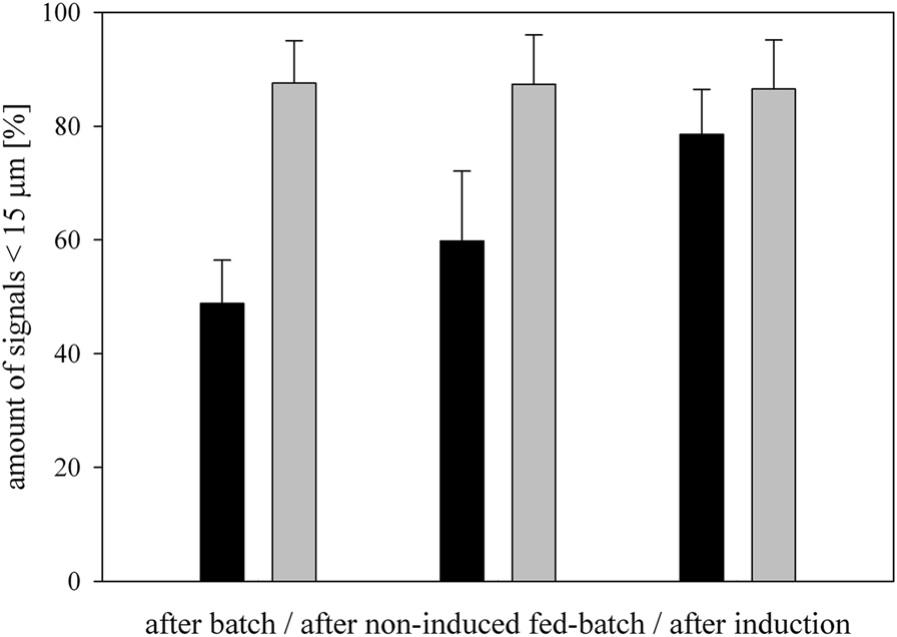
**Amount of cell signals < 15 μm measured at different time points during fed-batch cultivation.** Black bars, Δ *och1* strain; grey bars, wildtype OCH1 strain.

As shown in Table 3, the size distribution of the cells/cell clusters at the chosen time points of the different DoE cultivations was quite similar. In fact, all 3 factors were not significant for cluster formation. In Figure 3 the average amount of signals < 15 μm of the Δ*och1* strain were compared to the signals of the wildtype OCH1 strain.

**Figure 4:**
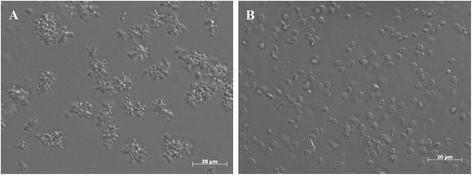
**Cell morphology after the fed-batch phase on glycerol of A, the recombinant Δ** ***och1*** **strain; B, the wildtype OCH1 strain.**

More than 80% of the signals for the wildtype OCH1 strain were smaller than 15 μm during the whole bioprocess (grey bars in Figure 3), whereas the Δ*och1* strain showed different size distributions dependent on the cultivation phase. After the batch on glycerol more than 50% of the signals indicated cell clusters. These clusters were still prominent after the glycerol fed-batch (40%). Only after switching the substrate to methanol, the clusters slowly disappeared. After 100 h of induction, around 80% of the signals were < 15 μm, which was similar to the wildtype OCH1 strain. Cell cluster formation was also followed by light microscopy. In Figure 4, typical Δ*och1* cell clusters [23] and budding cells for the control strain after the glycerol fed-batch are shown.

Apparently, cell cluster formation of the recombinant Δ*och1* strain was a function of the C-source and not of any of the 3 investigated process parameters. When grown on methanol, cell clusters disappeared over time. However, Δ*och1* cells stayed much smaller than wildtype OCH1 cells (Figure 4) which might result from the altered cell wall composition with enhanced chitin deposition [23].

#### Effects on cell lysis

Since we had observed intensive foam formation for the recombinant Δ*och1* strain before [23], which describes a huge problem for potential scale up, we monitored cell lysis by measuring extracellular DNA content over time. In Table 4 the extracellular DNA contents at the end of the different cultivations are shown. Although the extracellular DNA content constantly increased over time, more than 80% of the final DNA amount was already present after the fed-batch on glycerol for all cultivations. This strongly indicated that cell lysis went along with cell cluster formation and was not affected by any of the 3 process parameters. We speculate that cells in the center of the clusters get limited in either nutrients or oxygen and thus lyse. Once these clusters disappear due to the switch from glycerol to methanol, cell lysis is diminished. Since none of the 3 process parameters affected lysis, we had to adapt the cultivation strategy during glycerol fed-batch elsewise to avoid extensive foam formation. Initially, agitation was fixed to 900 rpm and the culture was aerated with 1.0 vvm dried air to guarantee dO_2_ values of > 30%. However, to avoid intensive foam formation, we decreased agitation to 600 rpm and aeration to 0.5 vvm, but added pure oxygen. Thus, although cells still lysed during glycerol fed-batch, intensive foam formation was avoided.

**Table 4:** **Extracellular DNA content measured at the end of cultivation**

**Cultivation**	**DNA content [ng∙μL** ^**−1**^ **]**
DoE1	451
DoE2	494
DoE3	519
DoE4	347
DoE5	485
DoE6	434
DoE7	339
DoE8	554
DoE9	429
DoE10	564

#### Effects on productivity and product purity

The effects of temperature, pH and dO_2_ on q_p_ (Additional file 1: Figure S1A), STY (Additional file 1: Figure S1B) and product purity (Additional file 1: Figure S1C) were analyzed and the respective data at the end of cultivation are summarized in Table 5.

**Table 5:** **Specific productivity (q**_p_ **), space-time-yield (STY) and specific enzyme activity at the end of cultivation**

**Cultivation**	**q**_**p**_ **[U∙g** ^**−1**^ **∙h** ^**−1**^ **]**	**STY [U∙L** ^**−1**^ **∙h** ^**−1**^ **]**	**Spec. activity [U∙mg** ^**−1**^ **]**
DoE1	3.30	151	61.7
DoE2	3.46	153	56.5
DoE3	0.03	0.45	0.21
DoE4	0.01	1.12	0.20
DoE5	0.06	1.35	0.29
DoE6	0.56	32	7.41
DoE7	3.49	137	47.2
DoE8	6.19	194	38.7
DoE9	0.96	39	15.9
DoE10	0.07	0.66	0.54

The only significant factor for all 3 responses was the temperature, whereas pH and dO_2_ had no effect (p-values for q_p_: temperature = 0.000072, pH = 0.77, dO_2_ = 0.33; p-values for STY: temperature = 0.000026, pH = 0.37, dO_2_ = 0.90; p-values for specific activity: temperature = 0.000024, pH = 0.49, dO_2_ = 0.45). The summary of fit plots indicated valid models (for q_p_: R2 = 0.94, Q2 = 0.88, Model Validity = 0.77, Reproducibility = 0.97; for STY: R2 = 0.95, Q2 = 0.81, Model Validity = 0.55, Reproducibility = 0.99; for specific activity: R2 = 0.95, Q2 = 0.93, Model Validity = 0.87, Reproducibility = 0.95). To visualize the effects, we plotted the responses versus temperature and dO_2_ at pH 5.0 (Additional file 1: Figure S1). Summarizing, both highest productivity and product purity were obtained at 20°C and a low dO_2_ at pH 5.0. Apparently, at lower temperature the recombinant Δ*och1* strain secreted more active HRP and less contaminating proteins compared to higher temperatures. This is not only important to increase the total amount of active product over time, but also for the subsequent downstream process. We observed a similar trend for the wildtype OCH1 strain where q_p_ and product purity were both higher at lower temperature: (20°C: q_p_ = 15 U∙g^−1^∙h^−1^, specific activity = 95 U∙mg^−1^; 24°C: q_p_ = 11 U∙g^−1^∙h^−1^, specific activity = 50 U∙mg^−1^; 28°C: q_p_ = 6 U∙g^−1^∙h^−1^, specific activity = 35 U∙mg^−1^).

### Electrophoresis and glycopeptides analysis by LC-ESI-MS

**Figure 5:**
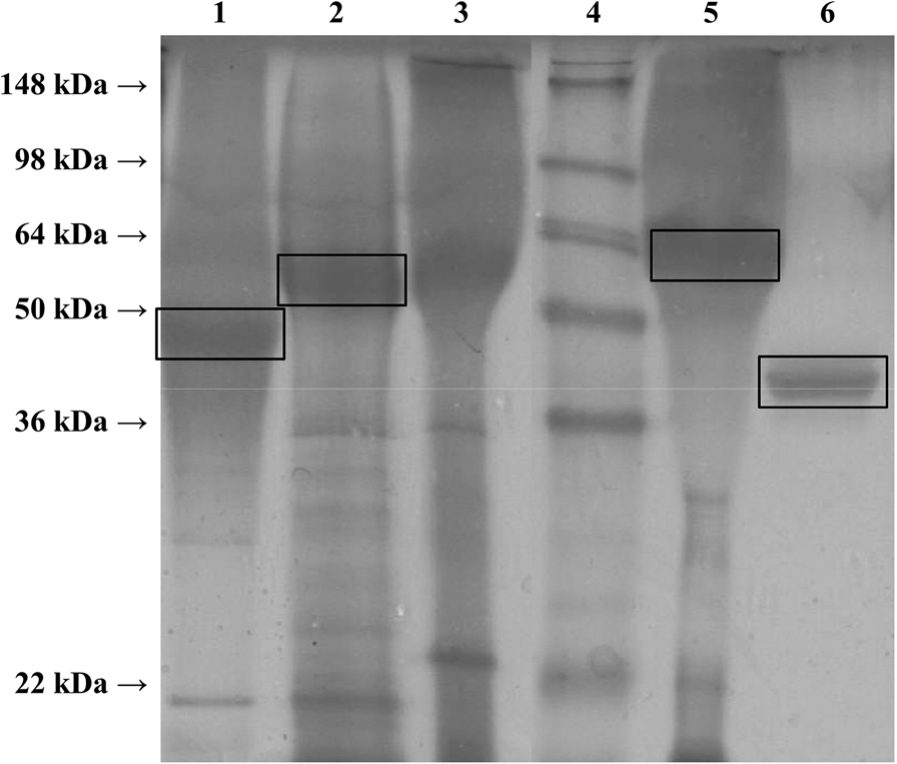
**SDS-PAGE gel of different HRP preparations.** Lane 1, cell free cultivation broth of cultivation DoE6 (HRP A2A from Δ*och1* strain); lanes 2 and 3, cell free cultivation broth of a wildtype OCH1 strain expressing HRP A2A; lane 4, SeeBlue*®* Plus2 Pre-Stained Standard (Lifetechnologies; Austria); lane 5, cell free cultivation broth of a wildtype OCH1 strain expressing HRP C1A; lane 6, commercially available HRP preparation from plant (P8375; Sigma; Austria).

Cell-free cultivation broths of the single fed-batch cultivations were analyzed by SDS-PAGE. Cell-free cultivation broth of a wildtype OCH1 strain as well as commercially available enzyme preparation from plant were included for comparison (Figure 5).

To identify the different HRP proteins, respective bands were excised (indicated in Figure 5) and glycopeptides were analyzed by HPLC-ESI-MS. In fact, we were able to identify the different HRP preparations (Additional file 2: Table S1). We observed apparent size differences between the 2 HRP A2A preparations from the Δ*och1* strain and the wildtype OCH1 strain, which might result from the different degree of glycosylation (lanes 1 and 2), and between HRP A2A and HRP C1A from the wildtype OCH1 strains (lanes 2 and 5; Additional file 2: Table S1).

### Verification runs at 15°C

The results of the DoE revealed a low cultivation temperature to be highly beneficial for productivity and product purity. To check if both responses could be further increased, 2 additional fed-batch cultivations at 15°C, pH 5.0 and dO_2_ of 10% were done. The average q_s MeOH adapt_ was 0.18 mmol∙g^−1^∙h^−1^, which was comparable to the results at 20°C and 25°C (Table 2). Apparently, significant changes in q_s MeOH adapt_ of the recombinant Δ*och1* strain only happened between 25°C and 30°C. Surprisingly, at 15°C productivity and product purity were only half compared to 20°C. We determined a q_p_ of 1.50 [U∙g^−1^∙h^−1^], a STY of 66 [U∙L^−1^∙h^−1^] and a specific activity of 23.3 [U∙mg^−1^] at 15°C, whereas in DoE2 (20°C, pH 5.0, dO_2_ 10%), q_p_ of 3.46 [U∙g^−1^∙h^−1^], a STY of 153 [U∙L^−1^∙h^−1^] and a specific activity of 56.5 [U∙mg^−1^] were measured. Thus, we concluded that the highest values for productivity and product purity, which are the main target variables in production processes, might be obtained between 15°C and 20°C, a pH of 5.0 and a dO_2_ of 10%.

## Conclusion

In the present study we developed a fed-batch bioprocess for a recombinant *P. pastoris* Δ*och1* strain. High productivity and product purity were reached when this strain was cultivated at pH 5.0, dO_2_ of 10% and a temperature of 20°C. At 30°C methanol accumulated over time due to apparent changes in cell metabolism. Cell cluster formation, which was accompanied by cell lysis, was dependent on the C-source. To avoid intensive foam formation during glycerol batch and fed-batch, aeration and stirrer speed had to be reduced.

Currently, we are investigating cell cluster formation on other C-sources, like glucose and sorbitol, and analyze the temperature range between 15-20°C in more detail to find the true optimum for the recombinant Δ*och1* strain. However, the present study already describes a good basis for bioprocess engineers working with glyco-engineered Δ*och1* yeast strains.

## Additional files

Additional file 1: Figure S1. Contour plot showing A, specific productivity; B, space-time-yield, and C, specific activity as an indicator for product purity at the end of cultivation in dependence on temperature and dO_2_. Additional file 2: Table S1. Results of the protein search with MASCOT (embedded in ProteinScape 3.0, Bruker) for protein identification of the excised bands. Only proteins identified with at least 2 peptides and a protein score higher than 80 were accepted. For searches the SwissProt database was used.
