# Supplementary figures and images for: Development of a fed-batch process for a recombinant Pichia pastoris Δoch1 strain expressing a plant peroxidase
Source: Microb Cell Fact. 2015 Jan 8;14:1. doi: 10.1186/s12934-014-0183-3 (PMC4335410; doi:10.1186/s12934-014-0183-3)

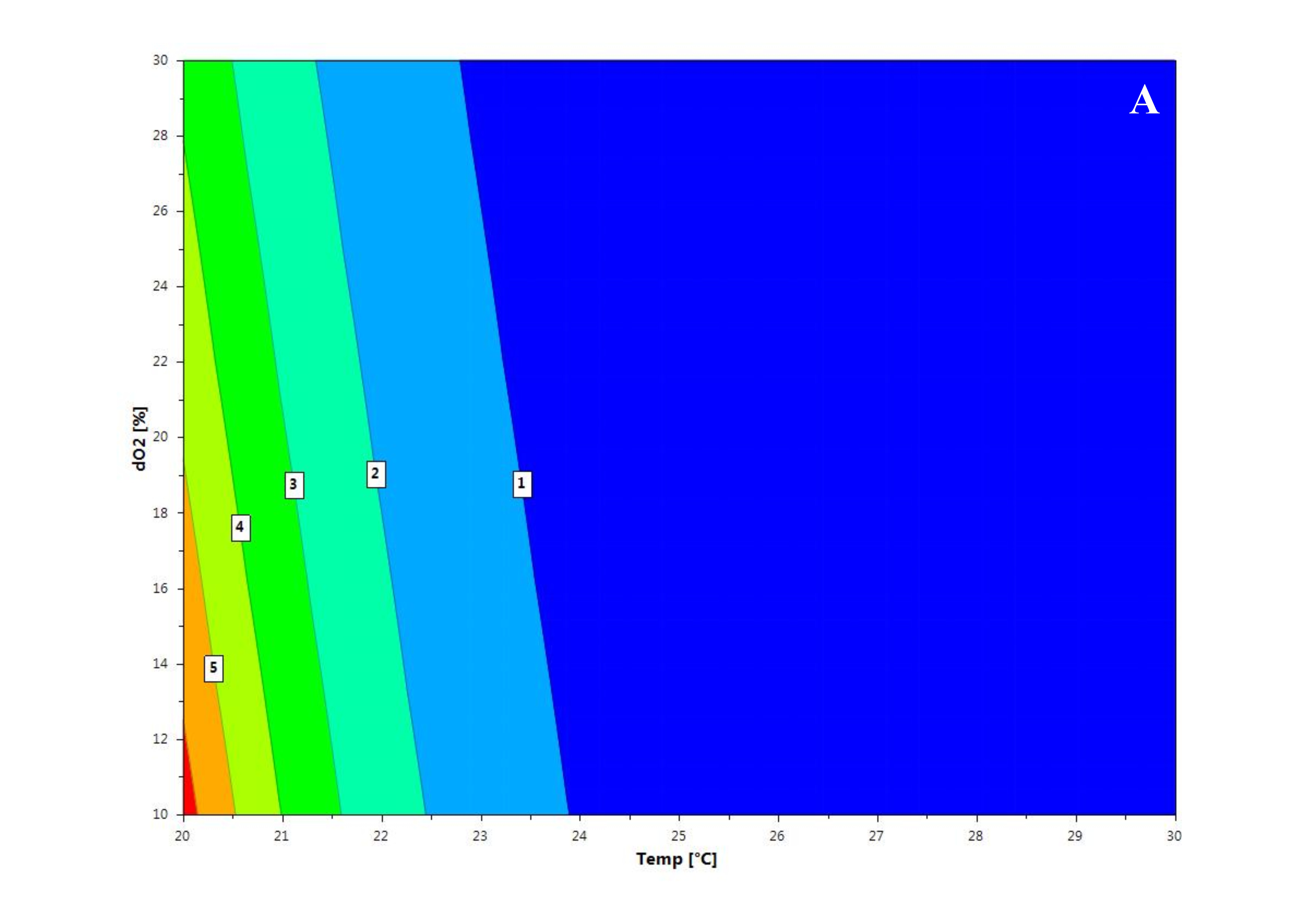

Supplement: Additional file 1: Figure S1. — Contour plot showing A, specific productivity; B, space-time-yield, and C, specific activity as an indicator for product purity at the end of cultivation in dependence on temperature and dO2. [file 12934_2014_183_MOESM1_ESM.zip › 12934_2014_183_add1a.jpeg]

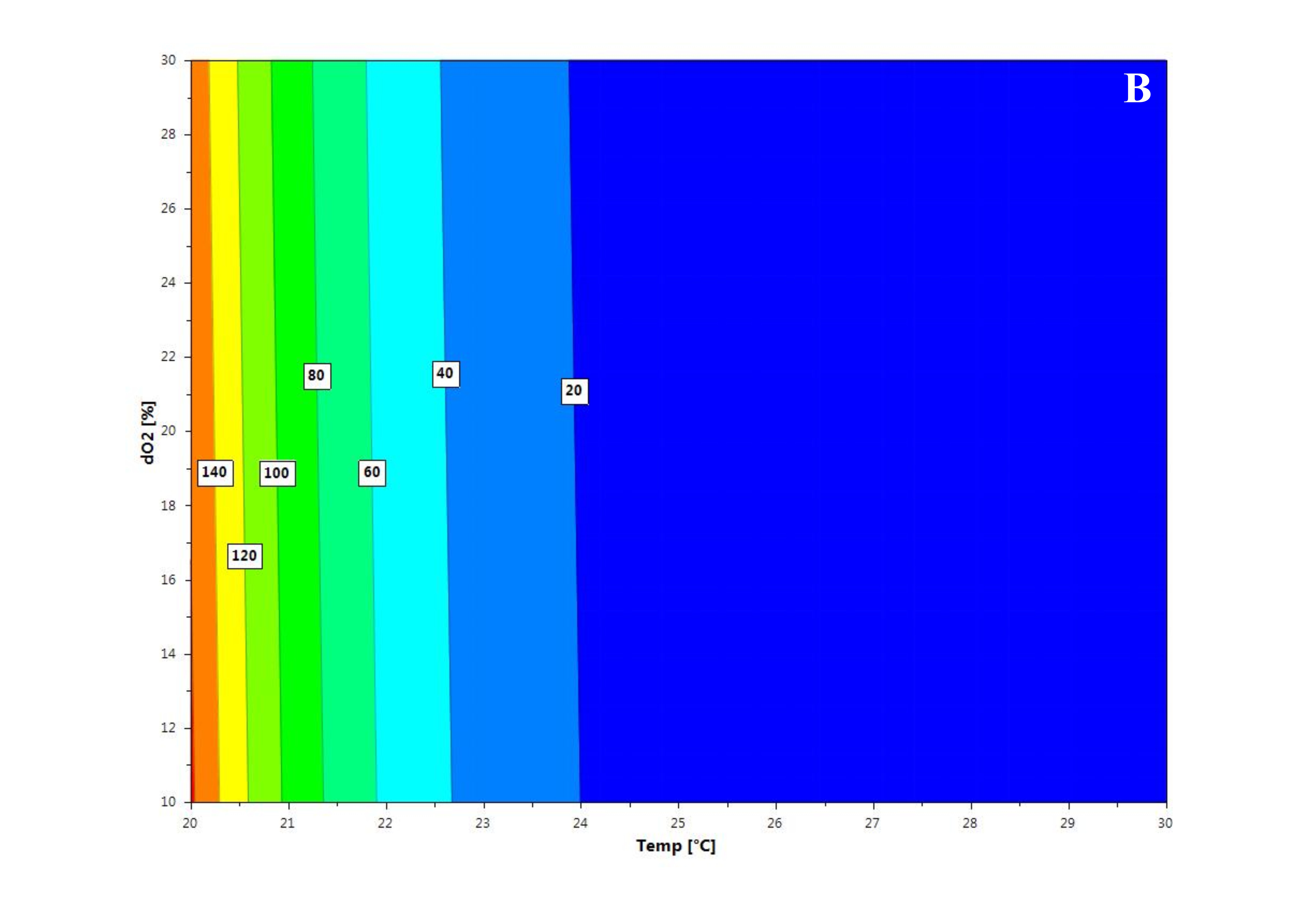

Supplement: Additional file 1: Figure S1. — Contour plot showing A, specific productivity; B, space-time-yield, and C, specific activity as an indicator for product purity at the end of cultivation in dependence on temperature and dO2. [file 12934_2014_183_MOESM1_ESM.zip › 12934_2014_183_add1b.jpeg]

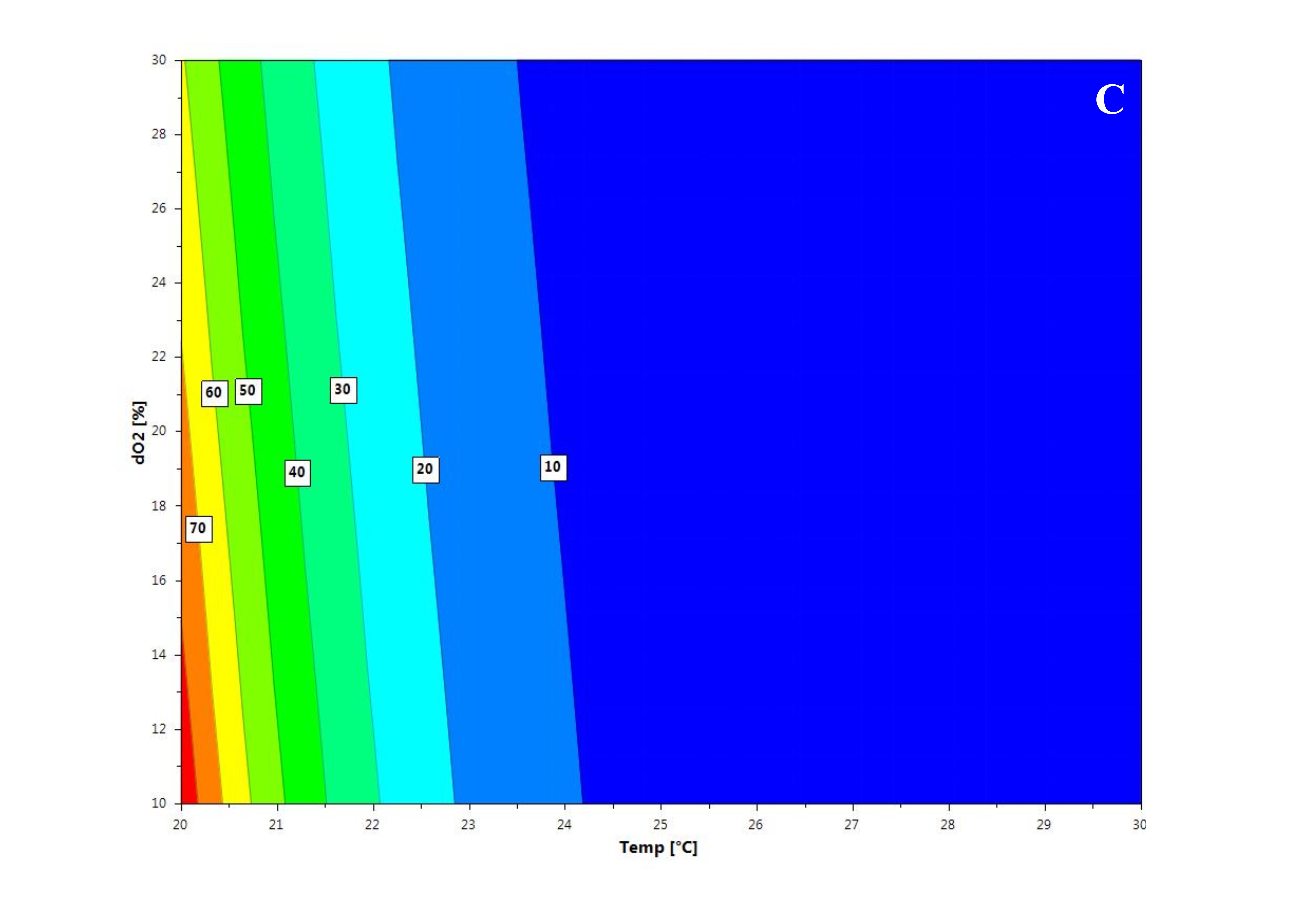

Supplement: Additional file 1: Figure S1. — Contour plot showing A, specific productivity; B, space-time-yield, and C, specific activity as an indicator for product purity at the end of cultivation in dependence on temperature and dO2. [file 12934_2014_183_MOESM1_ESM.zip › 12934_2014_183_add1c.jpeg]
