# Supplementary material for: Development of a fed-batch process for a recombinant Pichia pastoris Δoch1 strain expressing a plant peroxidase
Source: Microb Cell Fact. 2015 Jan 8;14:1. doi: 10.1186/s12934-014-0183-3 (PMC4335410; doi:10.1186/s12934-014-0183-3)
Supplement: Additional file 2: Table S1. — Results of the protein search with MASCOT (embedded in ProteinScape 3.0, Bruker) for protein identification of the excised bands. Only proteins identified with at least 2 peptides and a protein score higher than 80 were accepted. For searches the SwissProt database was used. [file 12934_2014_183_MOESM2_ESM.doc]

A**dditional file 2: Table S1** Results of the protein search **with MASCOT (embedded in ProteinScape 3.0, Bruker) for protein identification of the excised bands. Only proteins identified with at least 2 peptides and a protein score higher than 80 were accepted. For searches the SwissProt database was used.**

|  | **Rank** | **Accession** | **Protein** | **MW** | **pI** | **Score** | **Peptides** |
| --- | --- | --- | --- | --- | --- | --- | --- |
|  |  |  |  | **[kDa]** |  |  |  |
| Lane 1 | 1 | PERA2_ARMRU | Peroxidase A2 OS = Armoracia rusticana | 31.9 | 4.6 | 228 | 5 |
|  | 2 | TRYP_PIG* | Trypsin OS = Sus scrofa | 24.4 | 7.7 | 121 | 3 |
|  | 3 | K2C1_PANTR* | Keratin, type II cytoskeletal 1 OS = Pan troglodytes | 65.4 | 8.5 | 66.1 | 3 |
| Lane 2 | 1 | PERA2_ARMRU | Peroxidase A2 OS = Armoracia rusticana | 31.9 | 4.6 | 198 | 6 |
|  | 2 | TRYP_PIG* | Trypsin OS = Sus scrofa | 24.4 | 7.7 | 70 | 2 |
| Lane 5 | 1 | PER1A_ARMRU | Peroxidase C1A OS = Armoracia rusticana | 38.8 | 5.6 | 530 | 14 |
|  | 2 | TRYP_PIG* | Trypsin OS = Sus scrofa | 24.4 | 7.7 | 165 | 4 |
| Lane 6 | 1 | PER1A_ARMRU | Peroxidase C1A OS = Armoracia rusticana | 38.8 | 5.6 | 468 | 12 |
|  | 2 | TRYP_PIG* | Trypsin OS = Sus scrofa | 24.4 | 7.7 | 88.2 | 2 |

* impurities due to sample preparation.
